# Stroke death in patients receiving radiation for head and neck cancer in the modern era

**DOI:** 10.3389/fonc.2023.1111764

**Published:** 2023-06-15

**Authors:** Sara J. Hardy, Sanjukta Bandyopadhyay, Hongmei Yang, Annalynn Williams, Abdi Gudina, Michael A. Cummings, Hong Zhang, Deepinder P. Singh, Yuhchyau Chen, Nimish A. Mohile, Michelle C. Janelsins, Michael T. Milano

**Affiliations:** ^1^ Department of Neurology, University of Rochester Medical Center, Rochester, NY, United States; ^2^ Department of Radiation Oncology, University of Rochester Medical Center, Rochester, NY, United States; ^3^ Department of Clinical and Translational Research, University of Rochester Medical Center, School of Medicine and Dentistry, Rochester, NY, United States; ^4^ Department of Biostatistics and Computational Biology, University of Rochester Medical Center, School of Medicine and Dentistry, Rochester, NY, United States; ^5^ Department of Surgery, Cancer Control, University of Rochester Medical Center School of Medicine and Dentistry, Rochester, NY, United States; ^6^ Wilmot Cancer Center, University of Rochester Medical Center, Rochester, NY, United States; ^7^ Department of Neuroscience, University of Rochester Medical Center, School of Medicine and Dentistry, Rochester, NY, United States

**Keywords:** head and neck (H&N) cancer, IMRT, radiotherapy, stroke, SEER, cause of death

## Abstract

**Objectives:**

Head and neck cancer is a common malignancy frequently treated with chemotherapy and radiotherapy. Studies have shown an increased risk of stroke with the receipt of radiotherapy, but data on stroke-related mortality are limited, particularly in the modern era. Evaluating stroke mortality related to radiotherapy is vital given the curative nature of head and neck cancer treatment and the need to understand the risk of severe stroke in this population.

**Methods:**

We analyzed the risk of stroke death among 122,362 patients (83,651 patients who received radiation and 38,711 patients who did not) with squamous cell carcinoma of the head and neck (HNSCC) diagnosed between 1973 and 2015 in the SEER database. Patients in radiation vs. no radiation groups were matched using propensity scores. Our primary hypothesis was that radiotherapy would increase the hazard of death from stroke. We also examined other factors impacting the hazard of stroke death, including whether radiotherapy was performed during the modern era when IMRT and modern stroke care were available as well as increased HPV-mediated cancers of the head and neck. We hypothesized that the hazard of stroke death would be less in the modern era.

**Results:**

There was an increased hazard of stroke-related death in the group receiving radiation therapy (HR 1.203, p = 0.006); however, this was a very small absolute increase, and the cumulative incidence function of stroke death was significantly reduced in the modern era (p < 0.001), cohorts with chemotherapy (p=0.003), males (p=0.002), younger cohorts (p<0.001) and subsites other than nasopharynx (p=0.025).

**Conclusions:**

While radiotherapy for head and neck cancer increases the hazard of stroke death, this is reduced in the modern era and remains a very small absolute risk.

## Introduction

Head and neck cancer is a common malignancy, accounting for about 900,000 new cancer diagnoses annually worldwide (1) and 66,000 in the United States (2). Over the last two decades, data from randomized trials have changed the treatment paradigm for head and neck cancer, emphasizing organ preservation using concurrent chemoradiation (3, 4). Radiation Therapy Oncology Group (RTOG) 91-11 demonstrated that concurrent chemoradiation significantly improved locoregional control and larynx preservation rates, leading to increased use of chemoradiation for larynx preservation and maintenance of swallowing function (5). Meta-analyses have confirmed the benefit of concurrent chemoradiation in this setting, supporting the widespread use of this paradigm (6, 7).

Multiple studies have noted increased carotid stenosis and stroke rates in patients with head and neck cancer who receive radiation therapy (8–13). However, strokes can range from small infarcts with little impact on quality of life to large strokes associated with significant morbidity and mortality. Stroke mortality is a major public health burden, ranked as the second leading cause of death worldwide, with an annual mortality rate of 5.5 million (14). Stroke mortality is an important metric to understand the impact of stroke on a patient population. However, there are little data on stroke mortality in head and neck cancer patients and whether radiotherapy impacts risk. Notably, long-term results from RTOG 91-11 showed increased deaths unrelated to cancer in patients who received concurrent chemoradiation (5). The potential contribution of stroke death to these deaths is unclear, particularly in the modern era, where multiple factors have changed, including radiation techniques and the patient population. It is unknown whether modern radiation methods impact the risk of death from stroke. However, recent data show that radiation dose impacts the risk of stroke (15), and IMRT for early-stage laryngeal cancer can be used to reduce radiation exposure of the carotid arteries (16). Additionally, studies have shown that a history of smoking is associated with increased risk of post-radiation carotid stenosis (17). Patients with HPV-mediated cancers, who can have little or no smoking history, may not experience the same degree of stroke risk after radiation therapy. Thus, the increasing incidence of HPV-mediated cancers of the head and neck (18, 19) could impact stroke risk after radiation treatment.

We analyzed the risk of stroke death in patients with head and neck cancer using the National Cancer Institute’s Surveillance, Epidemiology, and End Results (SEER) Program registries using a propensity score-matched competing risk analysis. Our primary hypothesis was that radiation treatment would increase the hazard of death from stroke in subjects with squamous cell cancer of the head and neck. We also examined other factors impacting the hazard of stroke death, including whether radiotherapy was performed during the era when IMRT and modern stroke care were available. We hypothesized that the hazard of stroke death would be decreased in the modern era.

## Materials and methods

### Data source and population

The SEER 18 registries were utilized to analyze frequency, treatment, and survival information for patients diagnosed with squamous cell carcinoma of the head and neck (HNSCC) between 1973 and 2015. The SEER 18 database includes data from 18 population-based cancer registries in 14 states, covering 28% of the United States population (20). Available data include patient demographics, primary tumor site, tumor size, regional and distant metastasis, treatment, and survival. Institutional review board approval was not required for this study as the SEER database is publicly available and omits patient identifiers.

The SEER*Stat 8.3.9 Software package (SEER, National Cancer Institute, MD, USA) was used to identify patients diagnosed with a primary cancer between 1973 and 2015 in the eighteen registries of the SEER program. Patients with HNSCC were defined using the International Classification of Diseases for Oncology, third edition (ICD-O-3), histology codes for squamous cell carcinoma (8070–8078), malignant histology, and subsite codes for head and neck cancer (tongue, salivary gland, floor of mouth, gum and other mouth, oropharynx, tonsil, hypopharynx, other oral cavity and pharynx, larynx, nose, nasal cavity, and inner ear, and nasopharynx).

**Figure 1:**
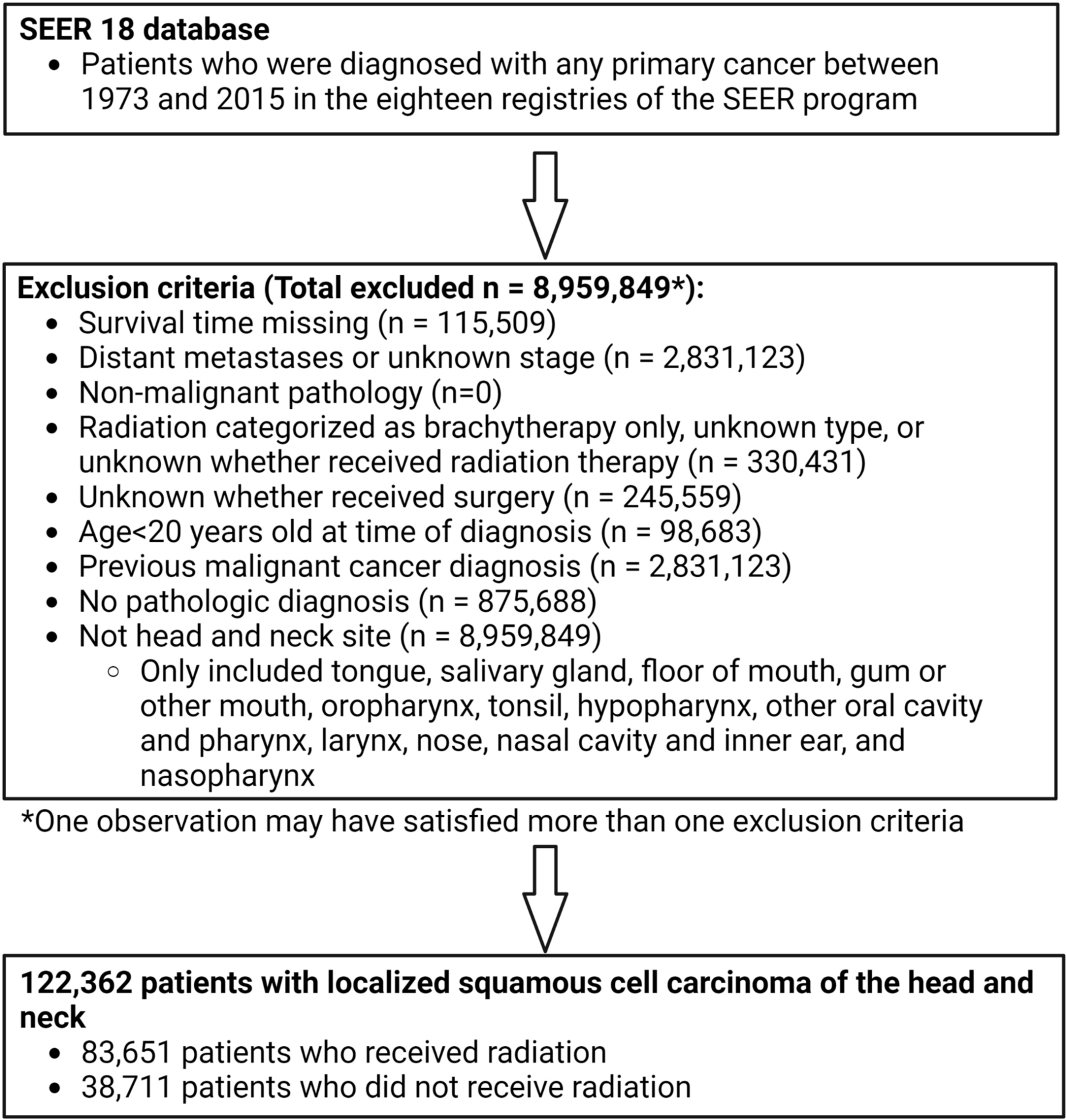
Participant inclusion flow chart showing inclusion and exclusion criteria applied to derive population used for propensity score matching. Patients diagnosed with any primary cancer between 1973 and 2015 were identified in the SEER 18 database. Multiple exclusion criteria were applied to derive a population of 122,362 patients with localized head and neck cancer. 83,651 of these patients received radiation and 38,711 did not receive radiation.

Patients were excluded if they did not have histologic confirmation of HNSCC. The stage at presentation was categorized according to the SEER historic stage A codes as ‘localized’ (localized without lymph node involvement or distant metastases, N0M0), ‘regional’ (locally advanced or lymph node- positive without distant metastases, N+M0), or ‘distant’ (distant metastases, any N M1). Patients with distant disease were excluded. Patients were only included if morphology behavior code was malignant. Patients were also excluded if 1) it was unknown whether or not they underwent surgery; 2) they received only non-external beam radiation (radioisotopes, radioactive implants); 3) it was unknown if they received radiation or what type of radiotherapy; 4) there were no data on follow-up or survival time (and therefore no information on the primary outcome of stroke death); 5) patients had a prior malignant primary cancer; or 6) age at the time of diagnosis was <20 years (**Figure 1**).

### Stroke-related death identification

Patients are categorized as alive or dead in the SEER database. Cause of death information is recorded and categorized into cancer-related and non-cancer-related cause of death. For our study, stroke death was defined as death from cerebrovascular diseases. For purposes of comparison, we also calculated rates of frequent causes of death in patients with head and neck cancer, including head and neck cancer death, other cancer death, cardiac death, chronic obstructive pulmonary disease (COPD) and allied conditions, pneumonia and influenza, suicide, all other known causes, and unknown causes.

### Treatment and covariate categorization

A dichotomous variable was generated for modern era, defined as diagnosis between 2001 and 2015, compared to non-modern era, those diagnosed between 1973 and 2000; IMRT was first used in the late 1990s, but utilization by radiation oncology practices increased dramatically starting in 2001 (21). Additionally, in 1995, recombinant tissue plasminogen activator (tPA) was demonstrated in clinical trials to improve neurologic outcomes from ischemic stroke, a major advance in stroke treatment (22).

While the SEER database is de-identified, county-level data on the population percentage with at least a high school education and median household income are available. Therefore, these factors were evaluated since socioeconomic status may impact the risk of death from stroke.

### Statistical analysis

Continuous variables are expressed as mean (+/- standard deviation; SD), while categorical variables are presented as absolute numbers and percentages. Homogeneity in both continuous and categorical variables between groups is assessed by standardized mean difference.

To minimize the selection bias for this cohort, propensity score matching (PSM) was used before estimating radiation effect on stroke-related survival with competing risk events (death due to other reasons). Logistic regression analysis with stepwise variable selection identified covariates associated with the use of radiation (*P* ≤ .05 for entry; *P* > .15 for removal). Next, logistic regression of radiotherapy assignment was modeled on selected baseline covariates (age, sex, race, marriage status, cancer grade and stage, and primary tumor site) that would otherwise confound comparisons between radiation groups to calculate propensity scores, which are the probabilities of getting radiation. Patients having radiation treatment and patients without radiation are matched using greedy nearest neighbor 1 to 1 matching (23). A standard caliper size of 0.5 difference between the logits of the propensity scores for the matched units was used. Standardized mean differences were estimated before and after matching to evaluate the balance of covariates; an absolute standardized mean difference less than or equal to 0.25 indicates a good variable balance between radiation groups (24, 25). The matched samples were used to explore the causal effect of radiation on stroke-related survival. The cumulative incidence function (CIF) of stroke-related death was generated using SAS version 9.4; deaths due to causes other than stroke were considered competing events. Gray’s test (26) was used to test the marginal effect (i.e., ignoring other predictors) of each potential predictor on stroke-related death. The proportional hazards assumption might not be appropriate for eras without IMRT use, despite the high significance of modern era use on CIF by Gray’s test (p < 0.001). Thus, the Fine and Gray competing risk model stratified by era was fitted using variables with p-values ≤0.15 by Gray’s test. In consideration of the pairing nature between the radiation group and the non-radiation group matched by propensity scores, the robust sandwich covariance formula was used together with the Fine and Gray subdistribution hazard model (27)

Furthermore, inverse probability treatment weighting (IPTW) was used on the entire population of eligible patients (n=122,362) before applying competing risk models to balance baseline covariates between the radiation groups for a safety check of the conclusions from the PSM method, which might lead to a significant reduction of sample size. The conclusions are the same between the bias-minimization approaches. The findings from the PSM are presented, while the results from the IPTW can be obtained upon request.

## Results

A total of 122,362 patients (83,651 patients who received radiation and 38,711 patients who did not) were identified based on the above criteria (**Figure 1**). Propensity score matching resulted in 31,492 pairs of matched subjects. The imbalance in all the demographics included for propensity score matching except grade was greatly reduced between radiation groups (**Table 1**, **Supplementary Figure 1**). Characteristics of the matched and unmatched populations are summarized in **Table 1**.

**Table 1:** Summary of baseline covariates and standardized mean difference between patients treated with radiation vs no radiation, before and after propensity score matching.

	Before propensity score matching (N= 122,362)					After propensity score matching (N=62,984)				
	Radiation: No		Radiation: Yes		Standardized Mean Difference**	Radiation: No		Radiation: Yes		Standardized Mean Difference**
	n	%	n	%		n	%	n	%	
**Surgery**					-0.751					-0.638
**No**	6,810	17.6	42,646	51.0		6,550	20.8	15,696	49.8	
**Yes**	31,901	82.4	41,005	49.0		24,942	79.2	15,796	50.2	
**Chemotherapy**					0.876					0.852
**No/Unknown**	36,470	94.2	50,763	60.7		29,297	93.0	18,811	59.7	
**Yes**	2,241	5.8	32,888	39.3		2,195	7.0	12,681	40.3	
**Race**					-0.076					-0.075
**Non-white**	5,420	14.1	14,042	16.8		4,549	14.5	5,416	17.2	
**White**	33,135	85.9	69,478	83.2		26,816	85.5	26,028	82.8	
Sex*					0.229					-0.083
**Male**	25,625	66.2	63,996	76.5		22,701	72.1	21,501	68.3	
**Female**	13,086	33.8	19,655	23.5		8,791	27.9	9,991	31.7	
Marital Status*					0.046					-0.008
**Married**	22,047	57.0	49,550	59.2		18,059	57.3	17,927	56.9	
**Unmarried**	16,664	43.0	34,101	40.8		13,433	42.7	13,565	43.1	
**Stage**					-0.660					-0.254
**Localized**	22,311	57.6	22,315	26.7		15,092	47.9	11,180	35.5	
**Regional**	16,400	42.4	61,336	73.3		16,400	52.1	20,312	64.5	
Grade*					0.330					0.517
**Low Grade**	25,395	65.6	43,631	52.2		19,500	61.9	12,893	40.9	
**High Grade**	6,691	17.3	25,426	30.4		6,484	20.6	13,850	44.0	
**Unknown**	6,625	17.1	14,594	17.4		5,508	17.5	4,749	15.1	
**Site**					0.601					0.490
**Tongue**	12,927	33.4	18,961	22.7		9,766	31.0	9,857	31.3	
**Salivary Gland**	701	1.8	1,326	1.6		660	2.1	1,009	3.2	
**Floor of Mouth**	5,129	13.2	4,295	5.1		2978	9.5	2,038	6.5	
**Gum and Other Mouth**	6,603	17.1	7,695	9.2		4835	15.4	3,281	10.4	
**Nasopharynx**	199	0.5	2,076	2.5		199	0.6	1,650	5.2	
**Tonsil**	2,556	6.6	16,053	19.2		2556	8.1	4,666	14.8	
**Oropharynx**	683	1.8	2,696	3.2		683	2.2	1,671	5.3	
**Hypopharynx**	1,379	3.6	6,078	7.3		1379	4.4	1,637	5.2	
**Other Oral Cavity and Pharynx**	463	1.2	1,607	1.9		463	1.5	994	3.2	
**Nose, Nasal Cavity and Middle Ear**	1,316	3.4	2,206	2.6		1218	3.9	1,059	3.4	
**Larynx**	6,755	17.4	20,658	24.7		6755	21.4	3,630	11.5	
**Treatment Era**					0.876					0.852
**2001-2015 (modern era)**	19,967	51.6	44,428	53.1		16176	51.4	18,380	58.4	
**1973-2000 (non-modern era)**	18,744	48.4	39,223	46.9		15316	48.6	13,112	41.6	
Age*					-0.162					-0.156
**20-39 years**	1,267	3.3	2,179	2.6		1190	3.8	1,603	5.1	
**40-59 years**	13,804	35.7	35,545	35.7		11594	36.8	13,558	43.1	
**60-84 years**	21,528	55.6	43,854	55.6		17174	54.5	15,126	48.0	
**85+ years**	2,112	5.5	2,073	5.5		1534	4.9	1,205	3.8	
**All**	38,711		83,651			31492		31,492		

variables with * are used in propensity score matching. ** Standardized mean difference is the mean difference divided by the standard deviation. An absolute standardized mean difference less than or equal to 0.25 indicates good variable balance between radiation groups.

To minimize the selection bias for this cohort, propensity score matching was used before estimating radiation effect on stroke-related survival with competing risk events (death due to other reasons). Standardized mean differences (the mean difference divided by the standard deviation) were estimated before and after matching to evaluate the balance of covariates.

Given the multiple competing causes of death for this patient population, cause of death data were examined. The most common cause of death was cancer death (26.797 patients, 42.5% of deaths, either head and neck cancer or other cancer death), cardiac death (5,153 patients, 8.2% of deaths), and COPD (1,585 patients, 2.5% of deaths). Stroke death was listed as the cause of death for 1,104 (1.8%) of the patients (**Supplementary Table 2**). Notably, there were 3.6 cases of stroke death per 1000 person-years in the non-modern era and 1.9 in the modern era (**Table 2**). Interestingly, cases of cardiac, COPD, and pneumonia-related death also decreased in the modern era (**Table 2**).

**Table 2:** Cause of death table comparing cases (per 1000 person-years) of stroke death to other causes of death including head and neck cancer, other cancers, cardiac death, COPD, pneumonia and influenza, suicide, and other.

	Modern era	Non-modern era
	Cases (per 1000 person-years)	Cases (per 1000 person-years)
**Cause of death**	45.5	38.5
**Head and neck cancer death**		
**Other cancer death**	31.7	27.0
**Cardiac death**	9.7	16.1
**COPD**	3.4	4.7
**Stroke death**	1.9	3.6
**Pneumonia and influenza**	1.2	2.6
**Suicide**	0.7	0.7
**Other**	15.3	16.7
**All**	109.4	109.8

Rates are shown separately for the modern era and non-modern era. Cause of death was significantly different by era (p<0.0001).

The hazard of stroke death was compared in the matched cohorts (radiation vs. no radiation). This hazard was significantly increased for patients treated with radiation therapy (p = 0.006, HR 1.203, CI 2.053-1.373). The population was not matched based on receipt of chemotherapy or surgery; however, surgery was not associated with a change in hazard of stroke risk (p = 0.453), but chemotherapy was associated with a reduced hazard of stroke death (p = 0.003, HR = 0.737, CI 0.603-0.901).

The effect of demographic and tumor subsite information on stroke death risk was also examined (**Table 3**). Female sex (p = 0.002, HR 1.221, CI 1.074-1.388), age group (p <0.001, HR 0.111, CI 0.061-0.202) for age 20-39 vs. 85+, 0.302, CI 0.235-0.387 for 40-59 vs. 85+, 0.594, CI 0.473-0.746 for 60-84 vs. 85+), nasopharynx subsite (p = 0.025, HR 1.733, CI 1.072-2.801) were significantly associated with hazard of stroke death. There was no significant association between treatment with surgery, race, county level income, stage, grade, county level percentage with less than high school education level, marital status, or other sites and hazard of stroke death.

**Table 3:** Subdistribution Hazard ratio of Stroke Death from the Fine and Gray competing risk model.

Parameter	Comparison Group	Hazard Ratio (95% Confidence Interval)	Pr > ChiSq
**Radiation**	**Yes vs No**	1.203 (1.053-1.373)	0.006
**Surgery**	**Yes vs No**	1.055 (0.917-1.214)	0.453
**Chemotherapy**	**Yes vs No**	0.737 (0.603-0.901)	0.003
**Marital status**	**Other vs married**	0.885 (0.780-1.004)	0.058
**Race**	**Non-white vs white**	1.016 (0.855-1.206)	0.859
**Sex**	**Female vs male**	1.221 (1.074-1.388)	0.002
**Cancer stage**	**Localized vs Regional**	1.032 (0.906-1.175)	0.634
**Histologic grade**	**High Grade vs Low Grade**	0.848 (0.727-0.988)	0.105
Age			
20-39 years	**vs age 85 or greater**	0.111 (0.061-0.202)	<.0001
40-59 years	**vs age 85 or greater**	0.302 (0.235-0.387)	<.0001
60-84 years	**vs age 85 or greater**	0.594 (0.473-0.746)	<.0001
**Median income (county)**		1.000 (0.994-1.007)	0.931
**Percent not completing high school (county)**		0.990 (0.979-1.001)	0.077
Head and neck cancer subsite			
Floor of Mouth	**vs other subsites**	1.191 (.0770-1.843)	0.432
Gum and other	**vs other subsites**	1.302 (0.854-1.986)	0.221
Hypopharynx	**vs other subsites**	0.668 (0.397-1.124)	0.128
Nasopharynx	**vs other subsites**	1.733 (1.072-2.801)	0.025
Oropharynx	**vs other subsites**	1.174 (.0702-1.962)	0.541
Salivary gland	**vs other subsites**	1.149 (0.671-1.967)	0.613
Tongue	**vs other subsites**	1.053 (0.694-1.598)	0.809
Tonsil	**vs other subsites**	1.160 (0.735-1.831)	0.524
Larynx	**vs other subsites**	1.332 (0.883-2.010)	0.172
Nose, Nasal Cavity, and Middle Ear	**vs other subsites**	1.462 (0.889-2.405)	0.135

**Figure 2:**
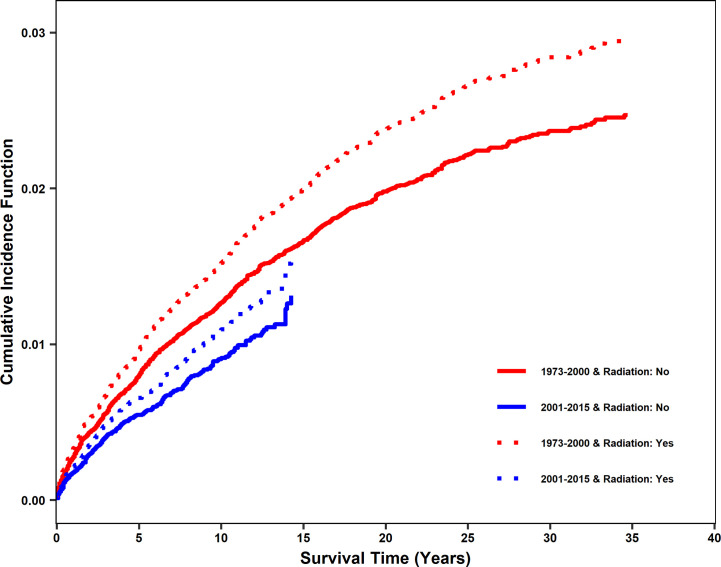
Cumulative incidence function of stroke death after diagnosis for patients treated with radiation vs no radiation in two eras: 2001-2015 (modern era) and 1973-2000 (non-modern era). In the propensity matched dataset, cumulative incidence function (CIF) curves for stroke death were generated for 2001-2015 and 1973-2000. Patients who received radiation and did not receive radiation are shown separately. CIF of stroke death was significantly reduced in the modern era. Patients receiving radiation are shown using dashed lines. Patients not receiving radiation are shown using solid lines. Patients diagnosed from 2001-2015 (modern era) are shown in blue and patients diagnosed from 1973-2000 (non-modern era) are shown in red.

The impact of radiotherapy on stroke death was compared for patients diagnosed between 1973 and 2000 (non-modern era, prior to contemporary radiation techniques, modern stroke care, and the increase in HPV-mediated cancers) and 2001 and 2015 (modern era). In the propensity-matched dataset, there were 817 incidences of stroke death between 1973 and 2000 and 287 between 2001 and 2015. The cumulative incidence function (CIF) of stroke death was found to be reduced in the modern era. However, for both eras, there was a significant increase in cumulative incidence of stroke death with radiation treatment (**Figure 2**), with CIF at 10 years being 0.009 (0.006, 0.013), 0.011 (0.008, 0.015), 0.013 (0.009, 0.017) and 0.015 (0.011, 0.020) for patients with no radiation and radiation in the modern era and with no radiation and radiation in the non-modern era, respectively, holding other variables constant. Diagnosis in the modern era significantly affected CIF by Gray’s test (p<0.001). However, it was included in the Fine and Gray competing risk model as a stratum since the proportional hazards assumption might not be appropriate due to modern radiation techniques, stroke care, and shifts in the head and neck population.

## Discussion

Based on propensity score matched competing risk analysis, our study showed that the hazard of stroke death was increased for patients who received radiation treatment for head and neck cancer. Other findings include nasopharyngeal cancer, larynx cancer, female sex, and treatment without chemotherapy as factors associated with greater hazards of stroke death.

Prior studies, including large population-based databases such as the Ontario Cancer Registry, have consistently shown an increased risk of stroke associated with head and neck radiotherapy (8, 9, 12, 15, 28–30). A recent meta-analysis found an increased risk of stroke in patients who received radiation therapy for cancer, with a relative risk of 2.09 (30). Cheng et al. performed carotid duplex imaging on 240 patients treated with head and neck radiation after a median follow-up of 6 years and found significant stenosis in 28 patients compared to 0 of 108 controls. Their multivariate analysis suggested that age >60 years, the interval from radiotherapy >5 years, and radiotherapy for either nasopharyngeal or laryngeal cancer were independent risk factors for developing significant stenosis (31)

While stroke risk is consistently increased, there are far fewer studies evaluating the impact of this risk of stroke on mortality. Stroke death is an important endpoint. While strokes can vary in severity and impact on quality of life, death is a concrete binary endpoint representing the most severe consequence of stroke. Swisher-McClure et al. demonstrated that patients with early-stage glottic cancer receiving radiation therapy have a higher risk of stroke death than those who underwent surgery alone (32). Notably, this study did not utilize propensity score matching to reduce effects from confounding factors to reduce selection bias, as healthier patients may be selected to undergo surgery and would also be less likely to die from stroke. As in our study, Chu et al. (8) demonstrated a greater risk of death from stroke in patients with nasopharyngeal cancer, which may reflect the standard practice of bilateral neck radiation for nasopharyngeal cancer. Our results show that while radiotherapy significantly increases the risk of stroke death in patients with head and neck cancer, the absolute risk among all patients is small (1.8%). By comparison, 42.5% of the head and neck patients in this analysis died of either head and neck cancer or another cancer (**Supplementary Table 2**).

In this cohort, cases of stroke death per 1000 person-years were reduced from 3.6 to 1.9 (a reduction of 47.2%) in the modern era. The hazard of stroke death also lower in the modern era. There are likely multiple reasons for these results. This may reflect some reduction in carotid dose exposure using modern radiation planning techniques such as IMRT and (more recently) image guidance and proton therapy. Long-term outcomes from Gupta et al. showed stroke toxicity only with 3D conformal radiotherapy (4). While historically, the carotid artery has not been contoured as an “object at risk” (OAR) to be spared using IMRT, more conformal radiotherapy delivery with IMRT coupled with the better ability to minimize hot spots using IMRT may decrease risks. Studies of carotid artery dosimetry with IMRT versus 3D plans have been limited to early glottic cancer, and further studies on how carotid artery dosimetry impact stroke risk are warranted. Further, with increasing awareness of long-term toxicities and use of IMRT, there has been a shift in practice towards unilateral treatment of cervical lymph nodes in some primary sites such as small well-lateralized tonsillar cancers, which could potentially reduce risk of stroke death. Additionally, the rising prevalence of HPV-related head and neck cancers may impact this finding (19). Smoking has been associated with higher risk of post-radiation carotid stenosis (17), and non-smokers with HPV-mediated cancers may not have the same increase in stroke-related death after radiation. Finally, major improvements in stroke care have occurred, particularly with the use of recombinant tissue plasminogen activator (tPA) in 1995 (22).

Also of note, patients treated in the modern era experienced less cardiac death, COPD-related death, and death from pneumonia with a reduction of 39.8%, 27.7%, and 53.8%, respectively (**Table 2**). This potentially reflects improvements in cancer care, better management of sequelae of head and neck cancer treatment, and changes in the head and neck population with higher prevalence of HPV-mediated cancers (18, 19). Patients with HPV-mediated cancers may have little or no smoking history (19); a reduction in smoking may at least partially account for the reduction in COPD, pneumonia, and cardiac death seen in the modern era, although modern treatments may be a factor as well.

The association of female sex with an increased hazard of stroke death in our study was unexpected as general population-based studies have shown that women experience more severe strokes but are less likely to die from stroke (33). Moreover, a small study showed that women were less likely to develop carotid stenosis after radiation therapy (34). More study is needed to understand the role of female sex in this outcome and whether this is related to systematic treatment or follow-up differences.

The lower risk of stroke death among patients who received chemotherapy may be attributable to radiation dose escalation or altered fractionation schedules in patients undergoing radiotherapy alone or possibly is a spurious finding attributable to confounding factors impacting patients selected to undergo chemotherapy or the under-reporting of chemotherapy use in SEER registries. A recent publication comparing SEER data with SEER-Medicare data reported that overall sensitivity was only 68% for SEER chemotherapy data (35). Other publications have reported that stroke risk is highest in patients with head and neck cancer who receive both chemotherapy and radiation. Additionally, chemotherapy is typically reserved for patients with better functional status and fewer comorbidities; the data on comorbidities within SEER are very limited. The presence of chemotherapy may also predict more aggressive disease and therefore death from cancer rather than death from stroke, especially in the modern era when concurrent chemoradiation is often given to HPV positive oropharyngeal patients, a population with lower rates of stroke risk factors like smoking. Finally, while we used a propensity score-matched population, patients were matched based on whether they received radiation, which may not have addressed confounding associated with chemotherapy use.

Strengths of the current study include the large, geographically diverse patient population representative of the US population, using a well-established and high-quality registry database. We also carefully designed this study and used appropriate methodology to control for potential sources of bias impacting stroke death through propensity score matching. Our results being derived from a large patient database with extended follow-up is essential given that stroke is a relatively uncommon consequence after treatment for head and neck cancer and may manifest many years after treatment, making it challenging to assess prospectively, particularly at single institutions.

Limitations of our study include its retrospective nature and the presence of unmeasured confounding factors that are not available in this SEER dataset, such as performance status, comorbidity index, smoking history, HPV status, which could correlate with head and neck cancer in non-smokers, and other factors. Other limitations include the lack of data on radiation dose and fields, particularly exposure of the carotid and intracranial vessels. Lastly, the SEER registries only allow tabulation of death from stroke and not the incidence of stroke. Certainly, the stroke rate would be much higher than the reported deaths from stroke, and patients who develop stroke may die from causes related to stroke but attributed to other causes in the death certificates.

Cerebrovascular complications from radiotherapy have been expected to rise over time due to cancer therapy advancements leading to longer patient survival times (29). However, modern radiation techniques, stroke treatments, and shifts in the head and neck patient population to include more HPV-mediated cancers will likely reduce the incidence of stroke death. This is particularly important given the improved prognosis seen in patients with HPV-mediated cancers (36). Our results suggest that while appropriate late effect surveillance and continued efforts to minimize radiation dose exposure to the carotids without compromising oncologic outcomes are important, the risk of stroke mortality is extremely low and this is important in understanding the long-term impact and benefits to patients with head and neck cancer receiving radiotherapy.

## Data availability statement

The datasets presented in this study can be found in online repositories. The names of the repository/repositories and accession number(s) can be found in the article/**Supplementary Material**.

## Ethics statement

Ethical review and approval was not required for the study on human participants in accordance with the local legislation and institutional requirements. Written informed consent for participation was not required for this study in accordance with the national legislation and the institutional requirements.

## Author contributions

SJH, MM, MJ: Conceptualization. SJH: Data curation and original draft writing. HY, SB: Statistical analysis. SJH, SB, HY, AW, AG, MC, HZ, DS, YC, NM, MJ, MM: Manuscript review and editing. All authors contributed to the article and approved the submitted version.

## Supplementary material

Supplementary Figure 1 Standardized mean differences in stage, marital status, sex, race, age, and primary tumor site between patients treated with RT vs no RT before matching and after matching, demonstrating reduction in baseline covariates with propensity score matching. To minimize the selection bias for this cohort, propensity score matching was used before estimating radiation effect on stroke-related survival with competing risk events (death due to other reasons). Standardized mean differences (the mean difference divided by the standard deviation) were estimated before and after matching to evaluate the balance of covariates; an absolute standardized mean difference less than or equal to 0.25 indicates good variable balance between radiation groups.
