# Supplementary figures and images for: Stroke death in patients receiving radiation for head and neck cancer in the modern era
Source: Front Oncol. 2023 Jun 15;13:1111764. doi: 10.3389/fonc.2023.1111764 (PMC10313411; doi:10.3389/fonc.2023.1111764)

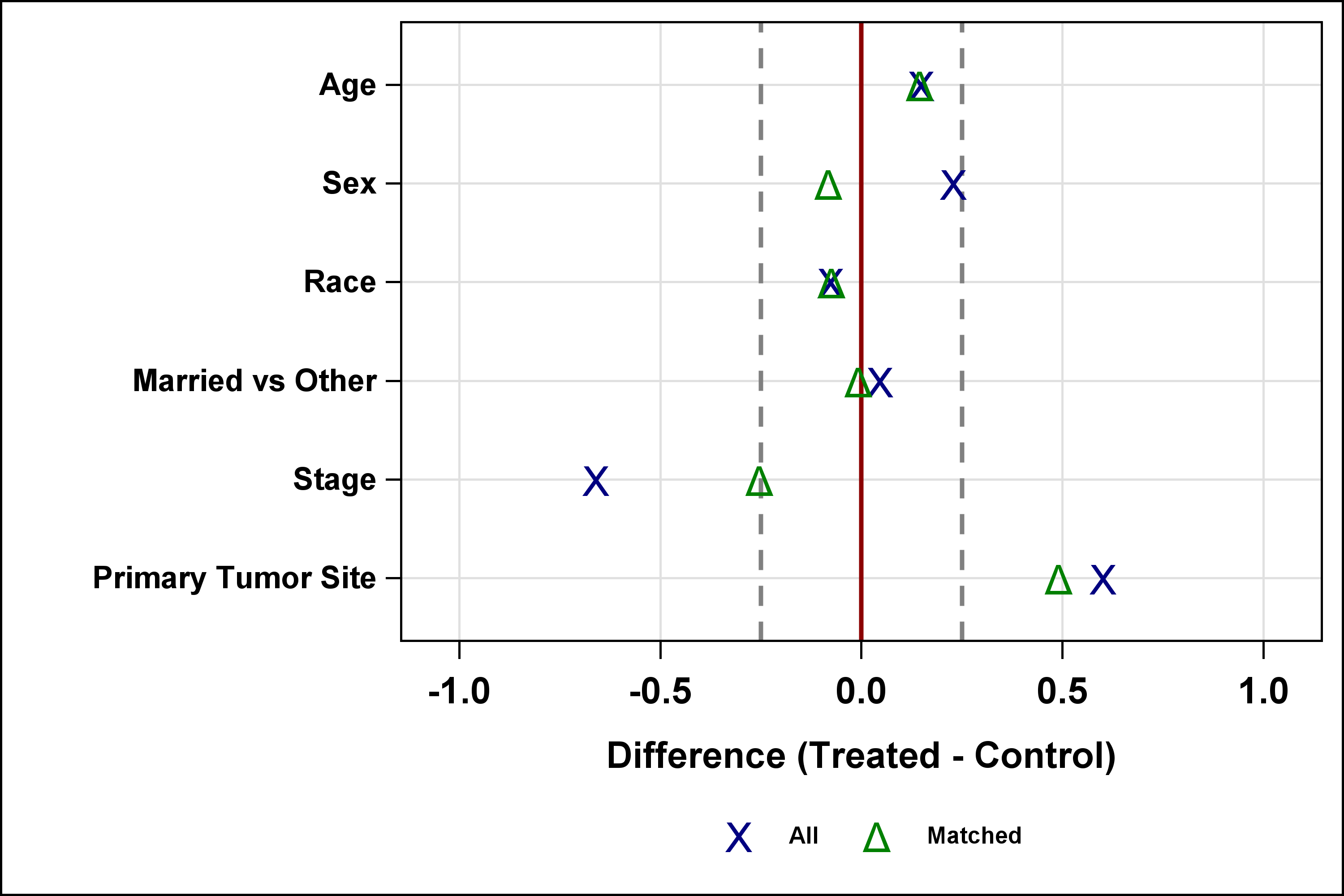

Supplement: Supplementary Figure 1 — Standardized mean differences in stage, marital status, sex, race, age, and primary tumor site between patients treated with RT vs no RT before matching and after matching, demonstrating reduction in baseline covariates with propensity score matching. To minimize the selection bias for this cohort, propensity score matching was used before estimating radiation effect on stroke-related survival with competing risk events (death due to other reasons). Standardized mean differences (the mean difference divided by the standard deviation) were estimated before and after matching to evaluate the balance of covariates; an absolute standardized mean difference less than or equal to 0.25 indicates good variable balance between radiation groups. [file Image_1.tif]
