# Supplementary material for: Stroke death in patients receiving radiation for head and neck cancer in the modern era
Source: Front Oncol. 2023 Jun 15;13:1111764. doi: 10.3389/fonc.2023.1111764 (PMC10313411; doi:10.3389/fonc.2023.1111764)
Supplement: Supplementary file 2 [file Table_1.docx]

**Supplementary Table 1. Results of matching patients treated with radiation vs no radiation by greedy nearest neighbor 1 to 1 propensity score matching.**

| **Propensity Score Information** | | | | | | | | | | | |
| --- | --- | --- | --- | --- | --- | --- | --- | --- | --- | --- | --- |
| **Observations** | **Treated (Radiation: Yes)** | | | | | **Control (Radiation: No)** | | | | | **Treated -**  **Control** |
|  | **N** | **Mean** | **Standard**  **Deviation** | **Minimum** | **Maximum** | **N** | **Mean** | **Standard**  **Deviation** | **Minimum** | **Maximum** | **Mean**  **Difference** |
| **All** | 83651 | 0.739 | 0.153 | 0.189 | 0.960 | 38711 | 0.565 | 0.207 | 0.189 | 0.960 | 0.174 |
| **Region** | 83651 | 0.739 | 0.153 | 0.189 | 0.960 | 38711 | 0.565 | 0.207 | 0.189 | 0.960 | 0.174 |
| **Matched** | 31492 | 0.688 | 0.196 | 0.189 | 0.960 | 31492 | 0.621 | 0.187 | 0.189 | 0.960 | 0.066 |
