# Supplementary material for: Stroke death in patients receiving radiation for head and neck cancer in the modern era
Source: Front Oncol. 2023 Jun 15;13:1111764. doi: 10.3389/fonc.2023.1111764 (PMC10313411; doi:10.3389/fonc.2023.1111764)
Supplement: Supplementary file 3 [file Table_2.docx]

**Supplementary Table 2.** Rates (number and percentage of total deaths) of stroke death and other causes of death including head and neck cancer, other cancers, cardiac death, COPD, pneumonia and influenza, suicide, other, and unknown.

| **Cause of death** | **Number** | **%** |
| --- | --- | --- |
| **Head and neck cancer** | 15,757 | 25.0 |
| **Other cancer** | 11,040 | 17.5 |
| **Cardiac** | 5,153 | 8.2 |
| **COPD** | 1,585 | 2.5 |
| **Stroke** | 1,104 | 1.8 |
| **Pneumonia and influenza** | 789 | 1.3 |
| **Suicide** | 262 | 0.4 |
| **Other** | 26,609 | 42.2 |
| **Unknown** | 685 | 1.1 |
| **Total** | 62,984 | 100.0 |
